# Comparison of constitutive and thiabendazole-induced expression of five cytochrome P450 genes in fourth-stage larvae of *Haemonchus contortus* isolates with different drug susceptibility identifies one gene with high constitutive expression in a multi-resistant isolate

**DOI:** 10.1016/j.ijpddr.2017.10.001

**Published:** 2017-10-07

**Authors:** Esra Yilmaz, Sabrina Ramünke, Janina Demeler, Jürgen Krücken

**Affiliations:** Institute for Parasitology and Tropical Veterinary Medicine, Freie Universität Berlin, Berlin, Germany

## Abstract

Benzimidazoles (BZs) remain amongst the most widely used anthelmintic drug classes against gastro-intestinal nematode infections, although their efficacy is increasingly compromised by resistance. The primary underlying mechanisms for BZ resistance are single-nucleotide polymorphisms (SNPs) in the isotype 1 β-tubulin gene causing the substitutions F167Y, E198A or F200Y. However, resistance is believed to be multi-genic and previous studies have shown that isolates carrying 90–100% F200Y can vary considerably in their resistance level in the egg hatch assay (EHA). Cytochrome P450 monooxygenases (CYPs) are associated with drug resistance in mammals and arthropods and have been considered as mediators of anthelmintic resistance. In *Caenorhabditis elegans*, several members of the CYP34/35 and CYP31 families are BZ and/or xenobiotic inducible and thiabendazole (TBZ) is metabolised by CYP35D1. Here, expression of all 5 CYPs closely related to the *C. elegans* CYP34/35 and CYP31 families was investigated in fourth-stage larvae of two susceptible and three BZ-resistant *Haemonchus contortus* isolates following *in vitro* exposure to TBZ for 3 and 6 h using real-time RT-PCR. The resistance status of all isolates was determined using EHAs and quantification of resistance-associated β-tubulin SNPs using pyrosequencing. While none of the CYPs was TBZ inducible, constitutive expression of CYP34/35 family member HCOI100383400 was significantly 2.4–3.7-fold higher in the multi-drug resistant WR isolate with the strongest BZ resistance phenotype compared to susceptible and intermediate-level BZ-resistant isolates. Although this increase is only moderate, HCOI100383400 might still be involved in high-level BZ resistance by further decreasing susceptibility in isolates already carrying 100% of a β-tubulin SNP causing BZ resistance. Lower transcript levels were observed for all CYPs in the intermediately resistant IRE isolate in comparison to the susceptible HcH isolate, which, except for CYP HCOI01579500, were statistically non-significant. This suggests that none of the investigated CYPs may contribute to protection against TBZ in this particular isolate.

## Highlights

• Comparison of 5 Cytochrome P450 expression in five *Haemonchus contortus* isolates.
• No thiabendazole-inducible expression of 5 Cytochrome P450 members.
• Increased basal expression of a Cytochrome P450 in a multi-drug resistant isolate.

## Graphical abstract

**Figure:**
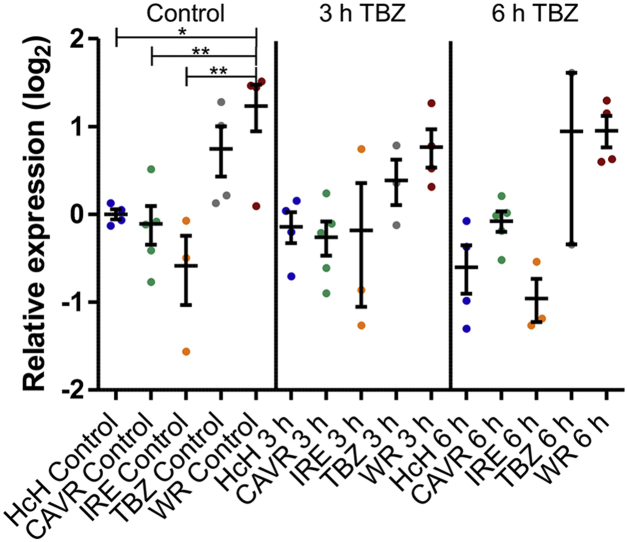

## 1 Introduction

In small ruminants, *Haemonchus contortus* is one of the most pathogenic gastrointestinal nematodes and infections with this parasite cause considerable economic losses (Perry et al., 2002, Peter and Chandrawathani, 2005). Treatment of *H. contortus* and other trichostrongyloids has become increasingly difficult due to the emergence of anthelmintic resistance, particularly in countries that heavily relied on use of anthelmintics in livestock production. Despite intensive research, anthelmintic resistance mechanisms are not fully understood yet. Recently, a study reported insufficient activity of the benzimidazole (BZ) albendazole against human *Ascaris lumbricoides* infections (Krücken et al., 2017).

Thiabendazole (TBZ) belongs to the class of benzimidazole (BZ) anthelmintics which have been in veterinary use against nematode infection since the 1960s. Resistance to BZs has been reported to emerge through single nucleotide-polymorphisms (SNPs) in the isotype 1 β-tubulin gene in codons 167, 198 and 200 in a number of trichostrongyloid nematodes (Grant and Mascord, 1996, Elard and Humbert, 1999, von Samson-Himmelstjerna et al., 2007, Skuce et al., 2010, Demeler et al., 2013, Ramünke et al., 2016) including *H. contortus* (Kwa et al., 1994, Prichard, 2001, Ghisi et al., 2007). However, no such SNPs in potential BZ target sites could be identified in the recent report on insufficient albendazole efficacy in *A. lumbricoides* (Krücken et al., 2017).

In addition to changes in the BZ target sites, non-target related alterations have been considered to be involved in anthelmintic resistance. In particular, drug efflux pumps, such as P-glycoproteins (Pgps), have also been addressed as possible mediators of anthelmintic resistance (Prichard, 2001, Janssen et al., 2013, Janssen et al., 2015, Kotze et al., 2014, Kaschny et al., 2015) – particularly against the macrocyclic lactones where no target-site changes have been associated with resistance. Regarding BZs, selection at the *pgp-2* locus in BZ-selected *H. contortus* has been described (Blackhall et al., 2008) and AlGusbi et al. (2014) have shown that the Pgp inhibitor verapamil potentiates the effects of TBZ on larval stages of BZ-susceptible and -resistant *Cooperia oncophora* and *Ostertagia ostertagi* leading to complete impairment of larval development. However, no activation of the rhodamine 123 (Rh123) binding site of Pgps and hence no increased efflux of Rh123 has been observed in eggs of a resistant *H. contortus* isolate following incubation with TBZ or albendazole while most macrocyclic lactones stimulated Pgp activity as revealed by significantly elevated efflux of rhodamine 123 (Kerboeuf and Guegnard, 2011). It is possible that BZs first need to undergo biochemical modifications to render them better substrates for efflux pumps. Moreover, *in silico* analysis of the binding properties of Pgp-1 of the free-living nematode *C. elegans* has predicted binding sites for TBZ and triclabendazole, though with less binding energy than drugs belonging to other classes of anthelmintics (David et al., 2016).

In recent years, the possibility of resistance via phase I (modifying) and phase II (conjugating) drug-metabolising enzymes and phase III transporters has received increasing attention. Regarding phase I, the largest group of drug-metabolising enzymes, the cytochrome P450 monooxygenases (CYPs), are known to be responsible for the biotransformation of a vast number of xenobiotics in mammals and insects and their association with insecticide and cancer drug resistance is well established (Rochat, 2005, David et al., 2013).

For a long time, it was assumed that CYPs exhibit no or very low activities in nematodes (Barrett, 1998). However, this general assumption has been weakened by a number of reports and attention devoted to CYPs is gradually increasing. The genome of *C. elegans* encodes more than 80 CYP proteins and several of these, in particular members of the family CYP35, have been shown to be inducible by xenobiotics (Menzel et al., 2001, Menzel et al., 2005). Increased expression of cyp35C1, cyp35A5 and cyp35A2 mRNAs has been reported in *C. elegans* upon albendazole exposure (Laing et al., 2010) and recently TBZ has been shown to induce *cyp35A3*, *cyp35A5*, *cyp35C1* and *cyp35D1* (Jones et al., 2013) and to be metabolised by CYP35D1 in *C. elegans* (Jones et al., 2015). Other CYP families that are inducible by xenobiotics are CYP34, CYP31 and CYP33 (Menzel et al., 2001).

In parasitic nematodes, studies on the xenobiotic response to BZs have progressed less far and focused mainly on *H. contortus*. One of the first reports on CYP activity was provided by Kotze (1997) using microsomal preparations of *H. contortus.* Consistent with phase I followed by phase II metabolism, *ex vivo* and *in vivo* studies have furthermore shown that in this parasite albendazole and fenbendazole are metabolised to albendazole- and fenbendazole-sulfoxide (Cvilink et al., 2008, Munguia et al., 2015). Also corroborating the hypothesis of CYP-mediated BZ resistance in gastro-intestinal nematodes, eggs and larvae of *C. oncophora* and *O. ostertagi* isolates with differing BZ susceptibility have been shown to become more susceptible to TBZ in the presence of the CYP inhibitor piperonyl butoxide (AlGusbi et al., 2014).

The CYP superfamily of *H. contortus* has recently been described in detail by Laing et al. (2015). They found that the CYP superfamily encoded in the *H. contortus* genome is considerably smaller than that of *C. elegans* and apparently lacks the extensive duplication of genes with function in metabolism of exogenous substrates. However, they identified four *H. contortus* CYPs that form a sister family to the xenobiotic-inducible *C. elegans* CYP families CYP34 and CYP35 (each with 10 members in *C. elegans*). This family will be designated as CYP34/35 family in the following. In *C. elegans*, one member of the CYP35 family, CYP35D1, has been shown to be inducible by TBZ and to contribute to detoxification of this anthelmintic (Jones et al., 2013, Jones et al., 2015). The *H. contortus* CYP34/35 family has apparently evolved through relatively recent gene duplications since HCOI01928800a and HCOI01928800b are located directly adjacent to each other on scaffold_930 in the *H. contortus* genome while HCOI100383700 and HCOI100383400 are separated by only two unrelated genes on scaffold_1500 (Laing et al., 2015). Other xenobiotic-inducible *C. elegans* CYP families have either no (CYP33) or only one gene (CYP31) in *H. contortus* (HCOI01579500) that has a corresponding position in the phylogenetic tree (see Fig. 3 in (Laing et al., 2015)).

Since CYP31A and CYP35D1 have been shown to be xenobiotic- and TBZ-inducible, respectively, and the latter was able to use the drug as substrate, this study aimed to investigate the expression patterns of the above mentioned, most closely related *H. contortus* CYPs in the fourth-stage larvae (L4) following *in vitro* exposure to TBZ using qRT-PCR. In order to determine any potential effects of unspecific (CYP-mediated) and specific (β-tubulin-mediated) resistance mechanisms, the current phenotypic and target-site related genetic level of resistance of five *H. contortus* isolates was determined by egg hatch assays (EHA) and pyrosequencing, respectively. For all three approaches, material from the same parasite passage was used.

## 2 Materials and methods

### 2.1 Parasites

Five isolates of *H. contortus* with differing susceptibility to BZs and other anthelmintics were incorporated in this study.

i) *H.c* Hannover (HcH, MHco9): susceptible to all anthelmintics.
ii) CAVR (MHco10) (Chiswick-Avermectin-Resistant): highly ivermectin (IVM) resistant, albeit BZ susceptible.
iii) IRE (inbred-resistant-Edinburgh; MHco5): highly IVM resistant; partially BZ resistant.
iv) TBZ (Thiabendazole): TBZ resistant; was originally obtained by Bayer Animal Health
v) WR (White River; MHco4): highly IVM and BZ resistant; moderately levamisole resistant

All isolates have been maintained at the Institute for Parasitology and Tropical Veterinary Medicine of the Freie Universität in Berlin for several years. Resistant isolates were regularly challenged by treatment of infected animals with the respective anthelmintic. All animal experiments were in agreement with the European directive 2010/63/EU and the German law (“Tierschutzgesetz”) and were approved by the responsible local authorities (LAGeSo Berlin) under the reference number L0088/10. Individual sheep were infected with approximately 6000 third stage larvae (L3) of one of the isolates and faeces were collected for egg recovery and larval cultures. For EHAs, only animals with at least 500 eggs/g faeces (epg) were used. Animals were treated orally with Cydectin 0.1% (0.2 mg/kg KGW) three weeks after begin of patency to clear the infections.

Eggs were purified from fresh faeces using a sucrose step gradient. Briefly, faeces were homogenised and passed through a 100 μm sieve. Eggs in the flow through were collected on a 25 μm sieve followed by centrifugation and flotation with saturated sodium chloride solution. Then, the egg suspension was laid on the top of a sucrose step gradient containing 10%, 25% and 40% of a saturated sucrose solution and centrifuged at 2000×g and 4 °C for 5 min. Eggs floated between the 10% and 25% layer. They were collected and washed with de-ionised water.

### 2.2 Egg hatch assay

Egg hatch assays (EHA) were principally carried out as described by Demeler et al. (2012). Recovered eggs were suspended in sodium phosphate buffer (10 mM NaPO_i_ buffer, pH 7) and adjusted to approximately 100 eggs/ml. TBZ (Sigma-Aldrich, T8904) was dissolved at a concentration of 10063 μg/ml in DMSO. From this stock solution, working solutions were prepared by dilution with DMSO. Stock and working solutions were prepared at least 24 h prior to use. Assays were set-up in 24-well plates with each well consisting of 1990 μl of egg suspension and 10 μl of drug solution or DMSO (vehicle control). A dilution range with final concentrations of 0.01, 0.024, 0.05, 0.077, 0.101, 0.157, 0.177, 0.201, 0.252 and 0.503 μg/ml of TBZ and positive control of 5.0315 μg/ml TBZ were used in assays with the isolates HcH, IRE and CAVR. For the highly resistant strains TBZ and WR, additional final concentrations of 0.75, 0.9, 1.0, 1.25 and 5.0315 μg/ml of TBZ and a positive control of 50.315 μg/ml TBZ were used.

Plates were incubated for 48 h at 25–27 °C and stopped with a drop of Lugol's iodine. All assays were performed at least in duplicate and repeated five times independently. Eggs and larvae were counted and the number of hatched larvae was calculated as percentage. EC_50_ values were determined by four parameter logistic regression using GraphPad Prism 5.0.3. Top constraints were restricted to values between 0 and 100% and bottom constraints were set to equal 0%.

Sums of square F tests were conducted to determine significant differences between isolates. All p values were corrected for multiple testing using the Holm correction as implemented in the p.adjust command in R statistics version 3.3.1.

### 2.3 DNA extraction and pyrosequencing of isotype 1 β-tubulin gene

Approximately 10,000 L3 of each isolate were concentrated using a Baermann Apparatus and kept at 80 °C until usage. DNA was isolated using the NucleoSpin Tissue XS kit (Macherey Nagel) according to manufacturer's instructions. To improve DNA yield, samples were initially thawed and homogenised in the presence of T1 Lysis Buffer (Macherey Nagel) using the SpeedMill P12 and innuSPEED Lysis Tubes F (both Analytik Jena) for three cycles of 1 min.

PCRs specific for *H. contortus* isotype 1 β-tubulin were run essentially as described by Ademola et al. (2015) by setting up 50 μl reactions containing 1 × HF Buffer, 1 U Phusion II High Fidelity DNA polymerase (Thermo Scientific), 0.2 mM dNTPs, 0.25 μM of each primer (HcPY2PCRFor: 5′-GACGCATTCACTTGGAGGAG-3′ and HcPY2PCRRev: 5′-Biotin-CATAGGTTGGATTTGTGAGTT-3′) and 2 μl of DNA (ca. 10 ng). Cycling conditions were as follows: initial denaturation at 98 °C for 30 s was followed by 40 cycles of 98 °C for 10 s, 56 °C for 30 s and 72 °C for 30 s. PCRs were terminated by a final elongation at 72 °C for 10 min. PCR products were used as templates for the subsequent pyrosequencing assays using the PyroMark Q24 system and the PyroMark Gold Q24 kit following the manufacturer's instructions. Sequencing primers used for measurement of allele frequencies were Hc167PySeq1: (5′-ATAGAATTATGGCTTCGT-3′), Haemcon198Seq-Pr: (5′-GGTAGAGAACACCGATG-3′), and Hc200PySeq1: (5′-TAGAGAACACCGATGAAACAT-3′).

### 2.4 *In vitro* cultivation of third to fourth stage larvae

The *in vitro* protocol for cultivation of *H. contortus* was modified from Rothwell and Sangster (1993) and Song (2003). Exsheathed L3s were obtained by incubating 30 ml of L3 suspension with 1 ml of 12.5% sodium hypochlorite for 10–15 min at 37 °C and 200 rpm. After microscopical control of exsheathment success, exsheathed L3 were washed twice with 0.9% NaCl solution by rapid mixing of larvae with solution and removal of solution by filtration through a bottle top sterile filter collecting the larvae on the top of the filter. Then, larvae were transferred to a Baermann Apparatus equipped with a 22 μm precision-woven nylon mesh and containing 0.9% NaCl solution. After incubation for at least 60 min, L3s were collected in a falcon tube and filled up with pre-warmed (37.5 °C) sterile axenisation fluid (136 mM NaCl, 5 mM KCL, 0.05 mM NaH_2_PO_4_, 0.1 mM Na_2_HPO_4_, 2 mM glucose, 0.024 mg/ml benzylpenicillin, 0.064 mg/ml streptomycin and 0.0008 mg/ml amphotericin B). Exsheated L3s were kept in this fluid at 37.5 °C and 90 rpm for 3 h. During this period, the axenisation fluid was replaced three times by removing the old solution by filtration on a bottle top filter.

Larvae were finally concentrated by filtration and maintained in pre-warmed RPMI 1460 culture medium (Lonza), supplemented with 20% foetal bovine serum (FBS) (Biochrom) and 0.06 mg/ml benzylpenicillin, 0.1 mg/ml streptomycin and 0.002 mg/ml amphotericin B, at a density of approximately 2080 larvae per ml culture medium at 41 °C and 20% CO_2_ for 2 days. On day 3, culture medium was replaced with fresh culture medium and larvae were incubated for another two days until exposure to TBZ.

### 2.5 Thiabendazole exposure of fourth stage larvae

Larvae were microscopically controlled to ensure that at least 80% had moulted to the L4 stage and displayed active pharynx pumping. Then, larvae were split into three groups of approximately 100,000 L4s each. Two groups were exposed to TBZ (0.5 μg/ml) for 3 h and 6 h, respectively. One group served as vehicle control and was incubated with 0.05% DMSO for 3 h. After incubation, L4s were washed with 4 °C cold 0.9% NaCl solution via filtration and collected in a falcon tube. L4s were pelleted through centrifugation at 4 °C and maximum speed for 5 min, re-suspended in cold 0.9% NaCl solution and again pelleted. The pellet was transferred to a lysis tube and frozen at −80 °C until used.

### 2.6 RNA extraction and cDNA synthesis

For optimal RNA isolation, larvae were rapidly thawed in the presence of T1 Lysis Buffer (Macherey Nagel) and homogenised using the SpeedMill P12 and innuSPEED Lysis Tube F (Analytik Jena) for three cycles of 1 min. The remaining steps were performed with the NucleoSpin RNA kit from Macherey Nagel according to manufacturer's instructions. Approximately 125 ng of RNA were treated with DNase I (Thermo Fisher) and reverse transcribed to cDNA using Thermo Fisher's Dynamo cDNA Synthesis Kit. For a 20 μl reaction mixture, 2 μl of M-MuLV RNase H+ reverse transcriptase and 1 μl of random hexamers were added to 10 μl RT buffer and RNA. cDNA was synthesised by incubating the mixture at 25 °C for 10 min, followed by 39 °C for 30 min and 85 °C for 5 min. Then, cDNA was diluted 1:3 and 1:6 for subsequent expression analysis of CYP- and reference genes, respectively.

### 2.7 Quantitative reverse-transcription PCR

Reference genes superoxide dismutase (*sod*), fatty acid retinol binding protein (*far*) and glyceraldehyde-3P-dehydrogenase (*gpd*) were adopted from Lecova et al. (2015) who had shown stable expression of these transcripts in adult *H. contortus* following varying treatments with albendazole and ivermectin. Primer sequences of both reference and candidate CYP genes can be found in Table S1 along with PCR efficiencies for each primer set. PCR reactions were run using the GoTaq qPCR Master Mix (Promega) on the CFX96 real-time PCR cycler (Biorad). A 50 μl reaction mixture was set up containing 25 μl of the GoTaq qPCR Master Mix for Dye-Based Detection, 21 μl of nuclease-free water, 0.2 μM of each primer and 2 μl of cDNA. Cycling conditions included initial denaturation at 95 °C for 2 min, followed by 40 cycles of 95 °C for 15 s and 60 °C for 1 min. A melt curve was generated by heating from 60 °C to 95 °C with increments of 0.5 °C each 5 s.

Samples were run in 2–5 biological and two technical replicates. All real-time PCRs were performed twice independently and primer efficacies were evaluated in every run via ten-fold serial dilutions with plasmid DNA carrying the respective genes (4 × 10^8^–- 4 × 10^1^ copies). Melt curves indicated absence of unspecific products. To account for run-to-run variation, a pool of plasmid DNA was used as a calibrator on each plate and data was analysed with the CFX™ Manager Software 2.0 which considers PCR efficiencies when calculating relative expression levels (Pfaffl, 2001). Statistical analysis to compare the gene expression level of target genes between treatment groups within one isolate and between different isolates without drug exposure was carried out using the one-way ANOVA with Tukey's post hoc test in GraphPad Prism 5.0.3.

## 3 Results

### 3.1 Phenotypic resistance status of isolates against thiabendazole determined by egg hatch assays

**Fig. 1:**
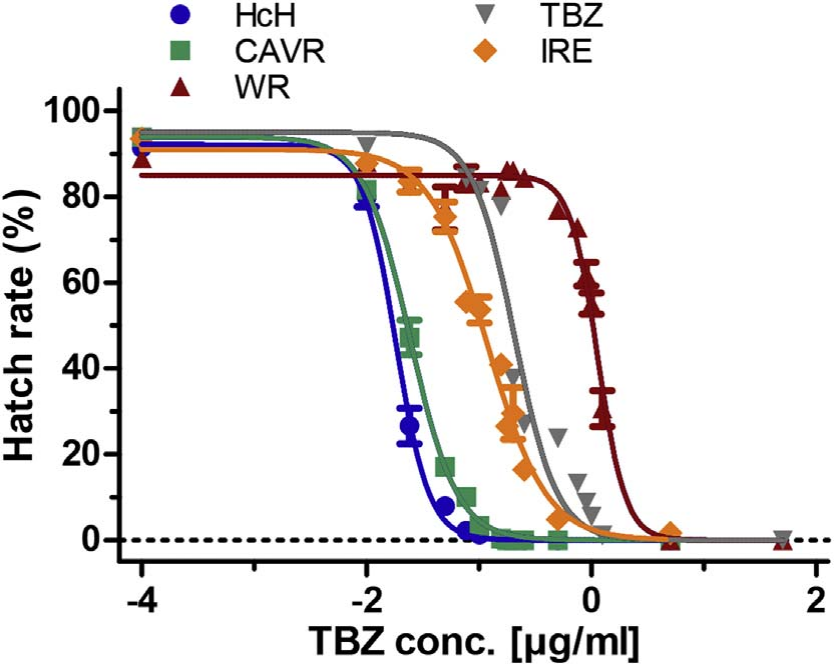
Concentration-response-curves for thiabendazole using different *Haemonchus contortus* isolates in an egg hatch assay. All EC_50_ values are significantly different from each other with a *p* = 0.0009.

Phenotypic resistance towards TBZ was determined by calculating EC_50_ values of all isolates. A wide range of EC_50_ values was found. For the BZ-susceptible HcH and CAVR isolates, EC_50_ values of 0.018 and 0.024 μg/ml, respectively, were obtained. With an EC_50_ value of 0.96 μg/ml, the WR isolate displayed the highest resistance towards TBZ. For the other two isolates of *H. contortus*, intermediate resistances were determined (EC_50_ values of 0.118 and 0.189 μg/ml for IRE and TBZ) (Fig. 1). The difference in EC_50_ values among all isolates were statistically highly significant (Table 1).

**Table 1:** Efficacy of thiabendazole against *Haemonchus contortus* in an egg hatch assay.

Isolate	EC_50_ [μg/ml]^a^	95% CI [μg/ml]^b^	R^2^^c^
*H.c* Hannover	0.01809	0.01708–0.01916	0.9787
*H.c* CAVR	0.02434	0.02293–0.02584	0.986
*H.c* IRE	0.1186	0.1054–0.1335	0.9448
*H.c* TBZ	0.1891	0.1817–0.1967	0.9399
*H.c* WR	0.9637	0.5814–1.597	0.9889

a Effective concentration 50%.

b 95% confidence interval.

c Coefficient of determination.

### 3.2 Frequency of benzimidazole-resistance associated single nucleotide polymorphisms determined by pyrosequencing

The frequency of the resistance-associated TAC SNP at codon 200 was strongly elevated in the BZ-resistant isolates WR (93.5 ± 2%, mean ± SD), TBZ (74.8 ± 2.7%) and IRE (64.5 ± 3.1%) compared to the BZ-susceptible HcH and CAVR isolates with frequencies of 6 ± 0.9% and 10 ± 1.4%, respectively, which corresponded to the technical background (Table 2). No evidence for increased frequencies of resistance-associated alleles at codons 167 and 198 wasfound, which is in agreement with previous results using the IRE and WR isolates (von Samson-Himmelstjerna et al., 2009).

**Table 2:** Mean frequencies (in %) ± standard deviation of benzimidazole resistance-associated alleles in the β-tubulin 1 gene.

Isolate	Codon 200 (TAC)	Codon 198 (GCA)	Codon 167 (TAC)
*H.c* Hannover	6 ± 0.9	13.5 ± 1	5.5 ± 0.6
*H.c* CAVR	10 ± 1.4	15.5 ± 1.7	10 ± 1.8
*H.c* IRE	64.5 ± 3.1	14.3 ± 1.5	6.3 ± 2.1
*H.c* TBZ	74.8 ± 2.7	13.2 ± 1.5	11.7 ± 1.3
*H.c* WR	93.5 ± 2	14.7 ± 1.3	2.6 ± 0.6

### 3.3 CYP expression in susceptible and resistant isolates of *Haemonchus contortus* following thiabendazole exposure

Due to insufficient *in vitro* development rates of L3 during the establishment phase of the cultivation method causing a limitation in available number of L3 and, more importantly, very low yield of RNA for some replicates, it was not possible to achieve five biological replicates for all isolates and time points. Since the experiments required large numbers of larvae (100,000 per replicate) and the number of available L3 was limited, no additional experiments were possible. For most isolates and experimental conditions, at least four replicates were available while only three were analysed for the IRE isolate and the TBZ isolate after 3 h exposure to TBZ and only two for the TBZ isolate after 6 h TBZ exposure.

**Fig. 2:**
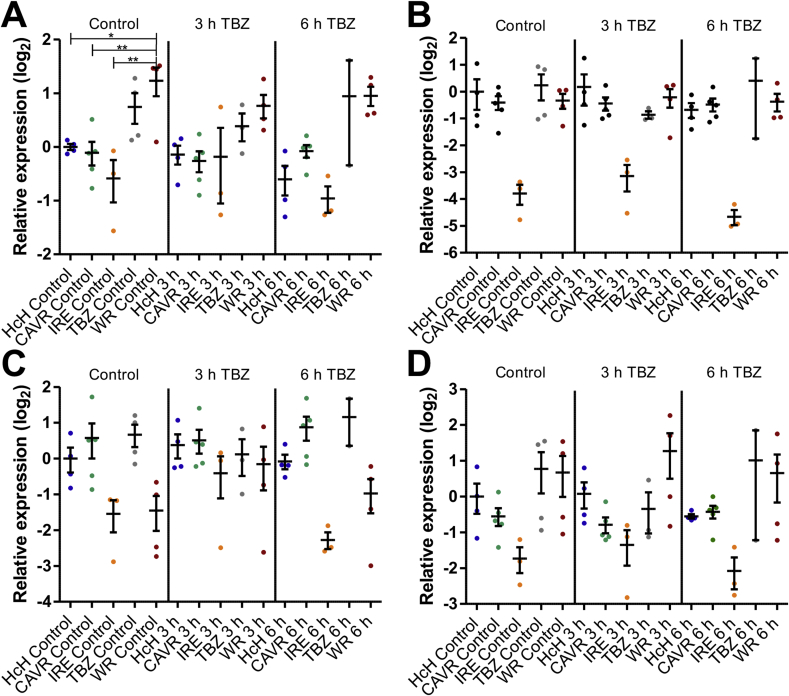
Expression levels of cytochrome P450 family 34/35 (Cyp34/35) mRNA orthologues in response to thiabendazole. Fourth stage larvae obtained by *in vitro* culture were exposed to 0.05% DMSO (vehicle control) for 3 h or 0.5 μg/ml thiabendazole (TBZ) in 0.05% DMSO for 3 or 6 h. Expression of the Cyps HCOI100383400 (A) HCOI100383700 (B), HCOI01928800a (C), HCOI01928800b (D) was analysed using the housekeeping genes superoxide dismutase (*sod*), fatty acid retinol binding protein (*far*) and glyceraldehyde-3P-dehydrogenase (*gpd*) as reference genes and the HcH isolate as control for normalisation.

**Fig. 3:**
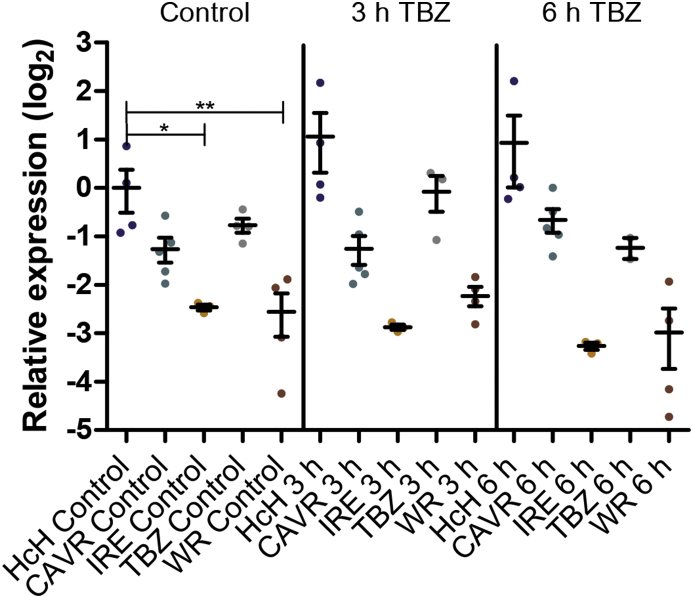
Expression levels of cytochrome P450 family 31 (Cyp31) mRNA orthologue in response to thiabendazole. Fourth stage larvae obtained by *in vitro* culture were exposed to 0.05% DMSO (for 3 h, vehicle control) or 0.5 μg/ml thiabendazole (TBZ) for 3 or 6 h. Expression of the Cyp HCOI01579500 was analysed using the housekeeping genes superoxide dismutase (*sod*), fatty acid retinol binding protein (*far*) and glyceraldehyde-3P-dehydrogenase (*gpd)*as reference genes and the HcH isolate as control for normalisation.

Transcript levels of investigated CYPs in all isolates showed no significant changes following 3 or 6 h exposure to 0.5 μg/ml TBZ when compared to worms of the corresponding isolate treated for 3 h with DMSO alone (Fig. 2, Fig. 3). However, only two biological replicates were available for the resistant TBZ isolate at the 6 h time point, which in case of CYP HCOI100383400, HCOI100383700 and HCOI01928800b showed a high degree of variability.

### 3.4 Comparison of basal transcript levels between isolates in the absence of drugs

Comparison of the basal transcript levels revealed no significant differences for HCOI100383700, HCOI01928800a, HCOI01928800b in any of the isolates (Fig. 2B, C, D). In contrast, significantly elevated transcript levels were observed for HCOI100383400 in the highly TBZ resistant WR isolate compared to both susceptible isolates, HcH (2.4-fold) and CAVR (2.7-fold), and the intermediately resistant IRE (3.7-fold) isolate (Fig. 2A). Transcript levels were slightly but not significantly elevated for the TBZ isolate. For HCOI01579500, transcript levels were significantly lower (approximately 5.7 fold) in the IRE and WR isolates compared to the HcH isolate (Fig. 3). The IRE isolate displayed lower transcript levels for the other CYPs as well when compared to the HcH isolate. However, these differences were not statistically significant.

## 4 Discussion

Cytochrome P450 monooxygenases have several times been considered as players in the evolution of anthelmintic resistance. However, the most convincing studies were conducted with *C. elegans* while knowledge of CYPs present in parasitic nematodes is still very scarce. For *H. contortus*, this has changed only recently, when Laing et al. (2015) characterised its CYP superfamily and provided a basis to assay specific CYPs. In the present study, five *H. contortus* isolates were evaluated regarding their resistance status by EHAs and pyrosequencing assays targeting codons 200, 198 and 167 of the isotype 1 β-tubulin gene. Then, the constitutive and TBZ-inducible expression of HCOI100383700, HCOI01928800a, HCOI01928800b, HCOI100383400 and HCOI01579500 were measured in L4 obtained by *in vitro* culture. The first four of the chosen CYPs are orthologues of the BZ- and/or xenobiotic-inducible CYP34 and CYP35 families in *C. elegans* while HCOI01579500 is an orthologue of the xenobiotic-inducible CYP31 family. Therefore, all closest phylogenetic relatives to the xenobiotic-inducible CYP families in the *H. contortus* genome have been included in the present study.

In vertebrates, the CYP gene superfamily is known to include so-called evolutionary stable and unstable genes. While stable CYP genes are typically the only members in their CYP family, are evolutionary conserved and function in biosynthesis and metabolism of endogenous substrates, unstable CYP genes generally show a high rate of gene duplication and deletion and encode enzymes acting on xenobiotic substrates (Thomas, 2007). The latter birth-death evolutionary scenario has been proposed to apply also to the evolutionary history of CYP genes in *C. elegans* (Thomas, 2007). Among the investigated *H. contortus* CYPs, HCOI100383700, HCOI01928800a, HCOI01928800b and HCOI100383400 all cluster in the same family, whereas HCOI0579500 appears to be a member of a CYP family containing no other members. Consistent with the above interpretation, all CYPs from the CYP34/35 family are more highly expressed in the intestine of adult *H. contortus* than in the soma (Laing et al., 2015). Since the intestine is hypothesised to be the major site of detoxification in nematodes (Lindblom and Dodd, 2006, McGhee, 2007, Rosa et al., 2015), this expression pattern would be in agreement with a role in detoxification.

Although exposure to TBZ did not induce a significant change in transcript level of any of the CYPs in the CYP34/35 family, basal expression of HCOI100383400 was significantly elevated in the highly resistant WR compared to the susceptible HcH, CAVR and the moderately resistant IRE isolates. However, the rather small difference in basal transcript level of this CYP in the resistant WR isolate suggests merely a minor role in resistance. For comparison, induction of *cyp35D1* by TBZ in *C. elegans* was 257-fold (1 h; 0.125 mM TBZ) and thus considerably higher albeit at a 50-fold higher TBZ concentration. Nevertheless, such a constitutive overexpression has the potential to protect against the effects of BZs at a very early time-point after exposure whereas inducible expression should be assumed to take several hours until enough functional enzyme has been synthesised to achieve considerable protection.

In this context, it should be noted that the WR isolate is resistant to IVM and also partially resistant to levamisole and increased expression levels of HCOI100383400 might also be a result of selection by these anthelmintics. However, in the absence of an inducibility by TBZ, investigations on potential effects of other anthelmintics on transcript levels and any functional data on drug binding or metabolism by HCOI100383400, it is impossible to assess which explanations are more likely. Another important aspect is that the isolates included in the study have completely independent genetic backgrounds. Thus, finding higher or lower expression level as for HCOI100383400 in WR and for HCOI01579500 in IRE and WR isolates, respectively is in no way conclusive. Differences between isolates might simply be by chance and completely independent of the resistance status.

The expression of the CYP31 orthologue HCOI01579500 is evidently higher in the soma of *H. contortus* than in the intestine and highest in adult females (Laing et al., 2015). Further, its *C. elegans* orthologues *cyp31A2* and *cyp31A3* are expressed in gonads and oocytes and have been shown to be indispensable for proper development of the embryo (Benenati et al., 2009). Even though there is no functional information available for HCOI01579500, the possibility of a similar critical function in development in *H. contortus* should not be completely excluded. Similarly, a role in xenobiotic metabolism should not be ruled out entirely, since CYPs may have activity against both endogenous and xenobiotic substrates as implied by the inducibility of *C. elegans cyp31A* by the xenobiotics PCB52, β-naphtoflavone, phenobarbital, atrazine, pyrazol and toluene (Menzel et al., 2001). Interestingly, the IRE isolate showed lower transcript levels for the other CYPs as well – all of which are expected to have exogenous substrates. However, these differences were non-significant which can most likely be attributed to the small number of biological replicates that were available for the IRE isolate since no other technical issues were encountered. In the absence of any known function of these CYP enzymes and considering that most differences were not significant it is not possible to conclude if lower expression levels in the IRE isolate might be physiologically relevant.

While the picture concerning the basal transcript levels of the five CYPs in the L4 stage of these isolates is clear, the picture concerning induced expression by TBZ is by far incomplete. The negative results obtained in the present study do not exclude that induction of CYP expression is possible under different experimental conditions. For instance, it cannot be excluded that an induction was not detected because up-regulation and return to basal levels might have occurred outside of the 3–6 h time window used here. Alternatively, CYPs might be responsive to TBZ in a concentration-dependent manner. Due to limitations in the number of larvae that were available, it was not possible to use a gradient of TBZ concentrations as previously done in the case of *C. elegans* for different albendazole concentrations (Laing et al., 2010). Here, a concentration of 0.5 μg/ml TBZ was chosen as a compromise aiming to use a concentration as high as possible while ensuring that susceptible isolates survive for the incubation time. Apart for a small number of individuals, larvae of both susceptible and resistant isolates were alive until the moment of incubation termination and the very few exceptions in all isolates most likely had no influence on the outcome of the analysis. However, due to the wide range of phenotypical BZ resistance within the five isolates, it might well be that the concentration window needed for induced expression of CYPs differs between isolates and that this window was missed. Another potential explanation for missing inducibility is the very artificial experimental setup. Since obtaining L4 in sufficient numbers from *in vivo* experiments is extremely difficult, *in vitro* culturing of L3 to L4 is a reasonable alternative to study responses of parasitic life cycle stages. Nevertheless, *in vitro* culture is a highly artificial system that might have unpredictable effects on the physiology of the L4s. Additionally, it remains to be elucidated whether constitutive expression of these CYPs is different in adult worms or other larval stages and whether expression can be induced by TBZ in adults or after *in vivo* treatment of the host.

Of course, the involvement of other CYPs that were not investigated here is also possible. This would be particularly plausible for genes belonging to rapidly evolving gene families with multiple members that have relatively recently evolved by gene duplication (Laing et al., 2015). This property again points to the four orthologues of the CYP34/35 family, as revealed by the analysis presented by Laing et al. (2015). Together, these data suggests that all members of rapidly evolving CYPs in the *H. contortus* genome have been included here. Nevertheless, the draft genome used to predict the *H. contortus* CYP gene family represents 93% of the conserved eukaryotic genes (Laing et al., 2013). Although this is a very good value for a nematode draft genome, the missing 7% suggest that also some of CYP gene superfamily members might have been missed.

In principle, mechanisms other than regulation of gene expression might be involved. Genome sequence analysis suggests that unstable vertebrate CYPs are subject to significant positive selection for amino acid changes possibly driven by xenobiotic substrates (Thomas, 2007). Although highly speculative, such a mechanism might be present in *H. contortus* or might eventually become relevant with the ongoing wide use of BZs. However, it cannot be excluded that all the CYPs that were investigated in the present study have no major function in xenobiotic mechanism at all. All currently available information provides only indirect arguments such as phylogenetic position, subfamily size and tissue distribution while direct evidence such as induction by and in particular metabolism of individual xenobiotics by particular CYPs is still completely missing for *H. contortus*.

The frequency of the resistance-associated SNP in codon F200Y was elevated in the phenotypically resistant IRE, TBZ and WR isolates. The rank order of the EC_50_ values in the EHA and the frequency of the resistance-associated SNP were identical over all five isolates investigated. This confirms that the genotype of the isotype 1 β-tubulin gene is the major parameter determining the BZ resistance status of *H. contortus*. However, comparison of the three phenotypically resistant lines identifies that a 10% increase in F200Y between the IRE (63.5% F200Y) and TBZ (73.5% F200Y) isolates is associated with an increase in the EC_50_ value by 1.6-fold. A further increase of the SNP frequency by 19% (92.5% F200Y in WR) is accompanied by a further increase in the EC_50_ value by 8.1-fold. Two phenomena might contribute to this dramatic additional increase. First, the frequency of homozygous worms carrying the allele associated with resistance is estimated to be 40.3% (IRE), 54.0% (TBZ) and 85.5% (WR) if random mating is assumed. Considering the technical background of pyrosequencing, data for the WR isolates do not even exclude that the isolate carries nearly exclusively the resistant allele. Nevertheless, it is hard to imagine that the dramatic increase in the EC_50_ value can be entirely attributed to increased frequency in F200Y. von Samson-Himmelstjerna et al. (2009) reported a F200Y frequency of 44% and an EC_50_ value in the EHA of 0.117 for the WR isolate. Since then, the isolate has gone through several *in vivo* selections using albendazole. While comparing EHA EC_50_ values for TBZ with F200Y frequencies of several isolates, von Samson-Himmelstjerna et al. (2009) identified eight isolates or field samples with F200Y frequencies between 90 and 100%. The EC_50_ values for these *H. contortus* populations ranged between 0.13 and 0.66 μg TBZ/ml – all lower than the WR isolate reported here. The high variability in phenotypic resistance between isolates with 90–100% F200Y suggests that selection at additional loci is able to further increase resistance levels. These might involve the isotype 2 β-tubulin gene (Kwa et al., 1993) but also other genes. If higher resistance levels are due to multi-genic effects, even moderate changes in constitutive expression of several genes could have considerable additive or synergistic effects on the resistance phenotype. In such a multi-genic context, constitutive or inducible overexpression of genes involved in detoxification of anthelmintics might occur only in isolates with the highest resistance level and different isolates with high resistance might differ in the set of overexpressed genes.

In conclusion, the results of the present study do not provide clear evidence that any of the *H. contortus* CYPs closely related to those involved in metabolism of xenobiotics or specifically TBZ in *C. elegans* has a role in resistance of the parasite. However, in combination with previous EHA and pyrosequencing data, the results also clearly show that (i) the isotype 1 β-tubulin gene locus cannot be the only one with influence on the phenotypic resistance level and (ii) that the *H. contortus* CYP gene HCOI100383400 should be considered as a candidate that might be involved in very high-level BZ resistance in a multi-genic context as well as in resistance to other anthelmintics.

## Conflicts of interest

The authors declare no conflicts of interest.

## Financial support

This work was financially supported by grant GRK 2046 from the German Research Foundation (DFG).
