# Supplementary material for: Comparison of constitutive and thiabendazole-induced expression of five cytochrome P450 genes in fourth-stage larvae of Haemonchus contortus isolates with different drug susceptibility identifies one gene with high constitutive expression in a multi-resistant isolate
Source: Int J Parasitol Drugs Drug Resist. 2017 Oct 7;7(3):362–9. doi: 10.1016/j.ijpddr.2017.10.001 (PMC5645160; doi:10.1016/j.ijpddr.2017.10.001)
Supplement: Supplementary material [file mmc1.docx]

**Table S1.**

**Primer sequences for quantitative reverse transcription PCR**

| Primer | Sequence | Amplicon length (bp) | Source | Mean PCR efficiency (in %) ± standard deviation |
| --- | --- | --- | --- | --- |

| Sod for | GCTGGCACTGATGATTTGGG | 73 | Lecova et al., 2015 | 95.7% ± 7.3 |
| --- | --- | --- | --- | --- |
| Sod rev | CGCCAGCATTTCCTGTCTTC |  |  |  |
| Gpd for | ACGAGACCTACAATGCAGCC | 67 | Lecova et al., 2015 | 90.4% ± 10.3 |
| Gpd rev | GCGAGACAGTTGGTGGTACA |  |  |  |
| Far for | TGCCAAGGACTATGCCAAGT | 128 | Lecova et al., 2015 | 88.6% ± 6 |
| Far rev | TGAGTGCGTCGATCTTTCCC |  |  |  |
| HCOI100383400 for | CGTTGCTGGACCTATGGTTT | 115 | Own design | 87.7% ± 5.5 |
| HCOI100383400 rev | AGCAACTCCTCTTCCACTCG |  |  |  |
| HCOI100383700 for | CAGCGATCTTCCCAATGAAT | 160 | Own design | 89.6% ± 5.1 |
| HCOI100383700 rev | TATCGGTCCGGATTGAACTC |  |  |  |
| HCOI01928800a for | CGACCAGGACCAAGCCC | 201 | Laing et al., 2015 | 88.5% ± 6.6 |
| HCOI01928800a rev | TCGCCTTGTTAGCTTCTTGAAA |  |  |  |
| HCOI01928800b for | TCGAACCAAGGAAGGCAAAC | 201 | Laing et al., 2015 | 89% ± 4.9 |
| HCOI01928800b rev  HCOI01579500 for  HCOI01579500 rev | CACCGAGACATGACCGTTTC  GCTGTGCATACTGTCAACGAT  CCTGCTCCTGGATCTCGC | 170 | Laing et al., 2015 | 82% ± 2.3 |
